# Positive Carotenoid Balance Correlates with Greater Reproductive Performance in a Wild Bird

**DOI:** 10.1371/journal.pone.0009420

**Published:** 2010-02-25

**Authors:** Rebecca J. Safran, Kevin J. McGraw, Matthew R. Wilkins, Joanna K. Hubbard, Julie Marling

**Affiliations:** 1 Department of Ecology and Evolutionary Biology, University of Colorado, Boulder, Colorado, United States of America; 2 School of Life Sciences, Arizona State University, Tempe, Arizona, United States of America; Stockholm University, Sweden

## Abstract

**Background:**

Carotenoids can confer somatic and reproductive benefits, but most evidence is from captive animal experimentation or single time-point sampling. Another perhaps more informative means by which to assess physiological contributions to animal performance is by tracking an individual's ability to increase or sustain carotenoids or other health-related molecules over time, as these are likely to be temporally variable.

**Methodology/Principal Findings:**

In a field study of North American barn swallows (*Hirundo rustica erythrogaster*), we analyzed within-individual changes in carotenoid concentrations by repeatedly sampling the carotenoid profiles of individuals over the course of the breeding season. Our results demonstrate that carotenoid concentrations of individuals are temporally dynamic and that season-long balance of these molecules, rather than single time-point samples, predict reproductive performance. This was true even when controlling for two important variables associated with reproductive outcomes: (1) timing of breeding and (2) sexually selected plumage coloration, which is itself positively correlated with and concomitantly changes with circulating carotenoid concentrations.

**Conclusions/Significance:**

While reproduction itself is purported to impose health stress on organisms, these data suggest that free-ranging, high-quality individuals can mitigate such costs, by one or several genetic, environmental (diet), or physiological mechanisms. Moreover, the temporal variations in both health-linked physiological measures and morphological traits we uncover here merit further examination in other species, especially when goals include the estimation of signal information content or the costs of trait expression.

## Introduction

Current research on carotenoids suggests that these molecules can improve health in a variety of animals, including humans [1], [2]. For example, circulating concentrations of carotenoids minimize oxidative stress [3], [4,], but see [5], and influence immune system activity [6]. Other studies suggest that circulating carotenoids are important underpinnings of condition-dependent signals related to mate-selection and competitive ability [7], [8]. However, many of these results have been obtained from animals in captive situations, where researchers have limited ability to assess the evolutionary and ecological context in which these molecules are linked to survival and reproduction. Moreover, previous analyses of carotenoids have relied on point-sampling methods whereas it is likely–though unknown–that an individual's carotenoid profile in nature is temporally dynamic, as circulating concentrations respond to daily nutritional supplies and to physiological demands for combating oxidative crises [9], [10], [11]. While these molecules certainly can be traded-off between somatic and reproductive functions [10], [12]–[14], the degree to which individuals sustain circulating carotenoid supplies and how these affect reproductive performance are unknown.

In a field study of breeding male and female barn swallows (*Hirundo rustica erythrogaster*) from North America, we assessed within-individual changes in carotenoid profiles as a function of both morphology and reproductive performance. We focused on carotenoids in our research because of previously established correlations between circulating carotenoids and fitness in this species [9], [15]. We also accounted for the degree of sexual ornamentation (ventral brown, melanin plumage coloration–not created by carotenoid pigmentation; [16]), which in European populations of barn swallows is associated with carotenoid status [15] and among North American birds is a strong predictor of social and genetic measures of breeding success [17], [18]. We predicted that individuals best able to maintain a positive balance of these health-associated molecules (likely via foraging efficacy) may also be capable of greater seasonal reproductive performance.

Our results suggest that single assessments of carotenoid status do not capture temporal variation in this health-related variable. Instead, a seasonal measure of *change* in carotenoid concentration is a strong predictor of annual reproductive success. Further, sexually-selected plumage coloration appears to be temporally related to changes in carotenoids: ventral color appeared to change concomitantly with circulating carotenoid concentrations.

## Materials and Methods

### Field Procedures

From May to August 2008, we monitored breeding in 71 uniquely marked adult barn swallows (33 males, 38 females) across 5 breeding sites in Boulder County, Colorado, USA (Latitude 40° 2′ 36″ N, Longitude 105° 16′ 39″ W). The number of breeding pairs at each site ranged from 8–18, (mean ± SD = 12±4.63). We captured birds at the start of the breeding season and obtained feather and blood samples as well as a number of morphological measurements, including the length of outer tail streamers and body mass. Swallows were recaptured for subsequent sampling throughout the breeding season. To compare changes in carotenoid concentrations across time, at three breeding sites we analyzed two successive plasma samples in 27 individuals for whom we had complete sets of morphological and reproductive performance data.

### Sample Collection

Plasma was collected from the beginning (6 May) to the end (8 August) of the breeding season, with most samples being collected during the first half of the breeding season (average Julian sample collection date ± SD = 164, or 12 June, ±22; mean Julian clutch initiation date ± SD = 149.7, or 29 May, ±10.7). Blood samples were kept on ice for a maximum of 1–3 hrs before centrifugation, at which point plasma was separated and stored at −80°C for 3 months prior to carotenoid analyses.

### Plumage Color Analyses

Following Safran & McGraw [17], plucked ventral feathers were taped to a standard white card background in the overlapping arrangement in which they appeared on the bird and stored in the dark. Feather samples were analyzed with an Ocean Optics USB4000 spectrometer (range 200–1100 nm) using a fiber-optic probe at an angle of 90° to the feather surface lit by a PX-2 pulsed xenon light source. Ambient light was excluded using a metal probe holder, which was placed against the feather sample; the probe was held at a constant distance from each sample, so that a 2.5 mm diameter of light hit the feather surface. Reflectance data were generated relative to a white standard (Ocean Optics WS-1) and a dark standard (all light excluded). Spectra were recorded with SpectraSuite software package (version 2.0.125), Ocean Optics Inc., Dunedin, FL). For each sample, 20 spectra were averaged to reduce noise from the spectrometer with an integration period of 200 ms. Our color measurement procedure was repeated three times for each sample, with the probe lifted between each scan. The three scans were performed at approximately the same location in the colored feather area. Repeatabilities were very high for our measures of color (R =  0.86–0.94), with brightness being the most repeatable (R =  0.94). The high repeatabilities of our color measurements indicate that the patch of color measured was indeed very homogenous, as we lift the sampling probe between measurements of the same sample. Further, measures among four ventral regions are also highly repeatable for this species [17] suggesting that our assessment of color in one region is fairly representative of color across the entire ventral surface of melanin-based feather coloration.

From the raw UV-VIS spectral data (300–700 nm), we computed tristimulus scores - hue, brightness, and chroma - which we defined as follows for the brown, melanic plumage of barn swallows [16]: hue is the wavelength of maximum slope for a brown, melanic trait, brightness is the total amount of light reflected by the feather surface across the range of considered wavelengths, and red chroma is the reflectance in the red color range (600–700 nm) divided by the total spectral range (300–700 nm). Because hue, chroma and brightness are highly intercorrelated for individual color patches [17], we chose throat brightness (brightness is our most repeatable color measurement) as our measure of color for both males and females. We considered using an integrated measure of color, like those obtained using principal components analysis [18], but because we examined changes in plasma carotenoids as a function of changes in color we needed to use absolute, as opposed to relative (transformed), measures of color.

### Carotenoid Assays and Analyses

We followed the methods of McGraw *et al*. [19] to extract carotenoids from 5–20 µl (mean ± SD = 18.54±3.66) plasma (following the ethanol: MTBE procedure) and to analyze carotenoid types and concentrations using high-performance liquid chromatography. Three carotenoid pigments were detected in plasma–lutein, zeaxanthin, and β-cryptoxanthin–all of which we previously reported in the egg yolks of barn swallows [20]. Concentrations were determined in ug/ml from external standard curves. Repeatabilities are very high for our measures of carotenoid and vitamin concentrations (R =  0.89–0.94; [see also 19]; n = 71). In both males and females, concentrations of two of three carotenoids were all highly and positively intercorrelated: (lutein-zeaxanthin, β-cryptoxanthin- zeaxanthin; Spearman rank rho: 0.64 and 0.38 respectively, both P<0.05; n = 71) with the exception of β-cryptoxanthin and lutein (Spearman rank rho: −0.26, P<0.05; n = 71). Because we needed a raw measure of carotenoid concentrations to quantitatively measure changes over time variable reduction methods (e.g. principal components analysis) is not appropriate. Accordingly, we summed levels of all three carotenoids to obtain total plasma carotenoid concentration.

### Statistical Analysis

We used mixed linear models to test for relationships between circulating plasma carotenoids with morphological and reproductive variables. These tests were run using JMP, version 7.0 software (SAS Institute, Cary, North Carolina). We incorporated ‘site’ as a random variable in our models to control for site differences and repeated measures of individuals at the same sites among our samples. We indicate this variable's significance in our tables by listing it in *italics* near the results of each model. All statistical tests reported are two-tailed and significance was assessed using an alpha level of 0.05. Degrees of freedom were calculated using the Kenward-Rogers method.

We directly analyzed gender differences in our data set in different ways. First (Table 1), we analyzed circulating concentrations as a function of morphological variation in each sex separately. Because we found similar effects of plumage color on carotenoid concentration in both males and females, we felt comfortable pooling individuals for each analysis to increase sample sizes for analyzing paired samples but included ‘gender’ (Tables 2 and 3) to conservatively control for possible sex differences in our data set. As no statistical effects of gender were detected, we did not include interaction terms with other covariates and ‘gender’ in multivariate models.

**Table 1:** Early season morphological correlations with circulating carotenoid concentrations in female (n = 38) and male (n = 33) barn swallows.

Variable	*Females*					*Males*				
	Estimate	SE	*ddf*	F	*P*	Estimate	SE	*ddf*	F	*P*
Streamer length (mm)	0.01	0.10	24.79	0.02	0.87	−0.07	0.07	22.96	1.12	0.30
Throat Color	−0.10	0.04	28	4.76	**0.04**	−0.11	0.05	13.49	5.35	**0.03**
Mass (g)	0.62	0.31	27.46	3.76	0.06	0.11	0.42	19.99	0.07	0.80

**Figure 1:**
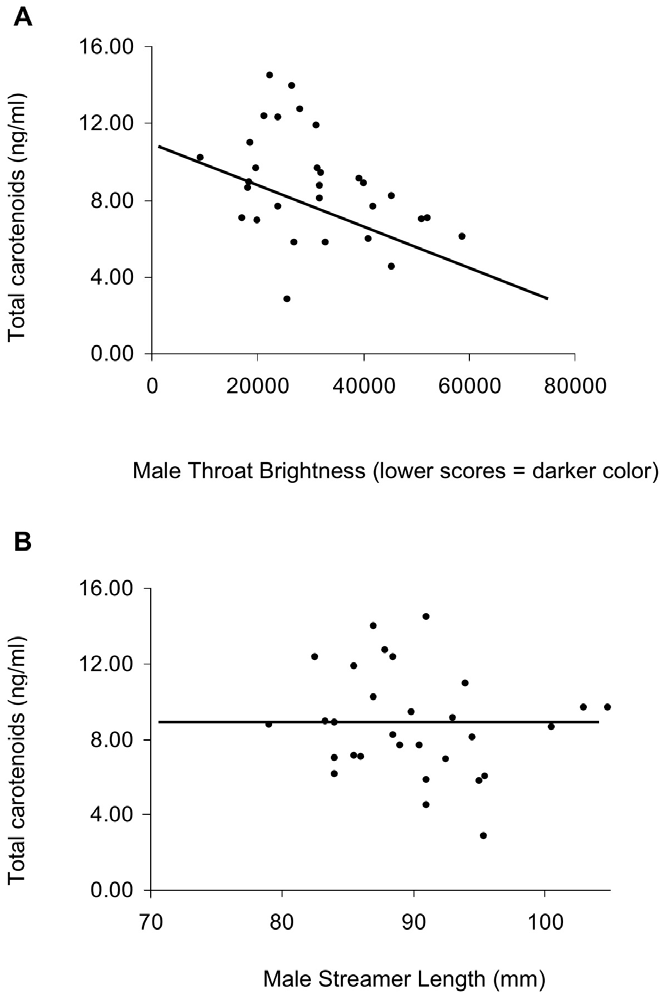
Circulating carotenoids are correlated with feather color, not tail streamer length. Variation in A) throat color, not B) streamer length, predicts variation in circulating carotenoid in male (shown) and female (similar trends not shown) barn swallows sampled at the beginning of the breeding season. N = 71 individuals.

Mixed models of total circulating concentrations of carotenoids in males (adjusted R-squared  = 0.15) and females (adjusted R-squared  = 0.34) in relation to morphology. Variation among breeding sites was controlled for as a random effect in each model. Numerator degrees of freedom  = 1, *ddf = denominator degrees of freedom* vary and are listed below. The negative relationship between throat color and carotenoids indicate that, in both males (Figure 1a) and females, darker individuals have greater concentrations of carotenoids at the start of the breeding season. Note that negative coefficient estimates between color and carotenoid concentrations indicate that less bright (visually darker) individuals have greater circulating concentrations of carotenoids during the early part of the breeding season.

**Table 2:** Controlling for possible gender differences, and site differences, individuals who maintained a positive balance of circulating carotenoids were greater in body mass.

Variable	Estimate	SE	*Ddf*	F	*P*
Gender	0.94	1.01	15.46	0.87	0.36
Streamer length (mm)	0.03	0.13	15.64	0.07	0.79
Throat Color	0.60	0.01	17.24	3.28	0.08
Mass (g)	0.56	0.25	17.41	4.74	**0.04**

Results portrayed are from mixed models of changes in carotenoids between two successive sampling events as a function of morphological measures in males and females. Adjusted R-squared  = 0.53. Numerator degrees of freedom  = 1, *ddf =  denominator degrees of freedom*. N = 27 individuals.

**Table 3:** Controlling for variation in breeding schedule (clutch initiation date), gender, and throat color, greater seasonal reproductive success is associated with positive carotenoid balance.

Variable	Estimate	SE	*ddf*	F	*P*
Clutch Initiation Date	−0.11	0.06	10.36	3.49	0.09
Gender	−1.32	0.74	10.67	3.19	0.10
Throat Color	−1.36	0.07	10.99	0.05	0.84
Seasonal change in carotenoids	0.77	0.34	9.338	5.00	**0.05**

Results portrayed are from a mixed model of seasonal reproductive success as a function of changes in carotenoids between two successive sampling events, controlling for gender and feather color. Adjusted R-squared  = 0.52. Body mass is highly correlated with changes in carotenoid balance (Table 2) and thus was not included as a covariate in this test, to avoid problems of multicolinearity. Numerator degrees of freedom  = 1, *ddf =  denominator degrees of freedom*. N = 27 individuals.

Because we employed a paired sampling design (repeated measurements on the same set of individuals), we calculated and then analyzed seasonal differences in several different variables, including mass, color, and circulating carotenoid concentrations. Within individuals, the differences between two successive samples were calculated by subtracting the first sample from the second. Accordingly, differences greater than zero indicate positive seasonal changes whereas differences less than zero indicate negative seasonal changes, unless specified, between sampling events. Moreover, in one analysis in which season-long measurements of all individuals in our study were pooled to examine differences in carotenoid concentrations across breeding sites, we use ‘individual’ as a random effect in the model to account for repeated measurements of individuals over time.

### Potentially Confounding Effects on Circulating Carotenoid Concentrations

Controlling for repeated samples of both males and females over time within the same set of breeding sites, we found differences in total carotenoid concentrations of individuals across five breeding sites (carotenoids as a function of site differences: F _4,72.99_ = 4.47, P<0.01, ‘individual identity’ controlled for as a random effect in the model *individual*) and as such controlled for these by using ‘site’ as a random variable in all analyses. Further, we found (1) no evidence of a seasonal decline in carotenoid concentrations (carotenoids as a function of sample date: F _1,53.95_ = 0.943, P>0.33, *site*), (2) no relationship between the sample amount used in assays and total circulating carotenoids concentrations (carotenoid concentration as a function of sample amount: F _1, 57.56_ = 0.04, P>0.80, *site*), and (3) finally, no influence of the number of days between sampling events (carotenoid concentration as a function of average number of days between samples ± = 33.40±19.27, range = 11–71; F _1,23.51_ = 1.70, P>0.20, *site*, n = 27) and total circulating carotenoids concentrations, thus justifying the exclusion of these potentially confounding covariates in our models.

In our analyses related to reproductive performance, we include the covariate ‘clutch initiation date’ because this is a strong predictor of the number of fledged young produced by pairs (earlier breeders have a higher probability of producing larger clutches and two sets of broods; [21], [22]), and as such controls for seasonal effects of lay date variation, food availability, and different breeding schedules. Finally, because we did collect repeated samples on individuals that ended up being socially paired at the same nest, we randomly removed pair-mates (n = 5) from our analyses to avoid pseudo-replication of reproductive performance data. The results are consistent in both the larger sample containing pair-mates and the reduced sample.

## Results

### Sex Differences in Melanin Plumage Color and Circulating Carotenoid Levels

As in our previous work [17], we found that males in our Colorado population had darker ventral coloration compared to females (throat brightness: F _1, 245.7_ = 23.22, P<0.001, *site*). Further, males had significantly greater concentrations of circulating carotenoids than females (F _1, 59.19_ = 28.88, P<0.001).

### Morphological Correlates of Carotenoid Concentrations and Balance

In the early part of the breeding season (first sampling event), feather color brightness and not streamer length or body mass, significantly predicted variation in total carotenoid concentrations in both males and females (Table 1, Figure 1), such that both males and females with darker plumage color (lower brightness scores) had greater circulating concentrations of carotenoids at the start of the breeding season.

Among morphological variables we analyzed, changes in carotenoid concentrations are predicted by body mass at the start of the breeding season, where heavier individuals (both males and females) were able to maintain greater carotenoid concentrations compared to birds lighter in mass (Table 2).

**Figure 2:**
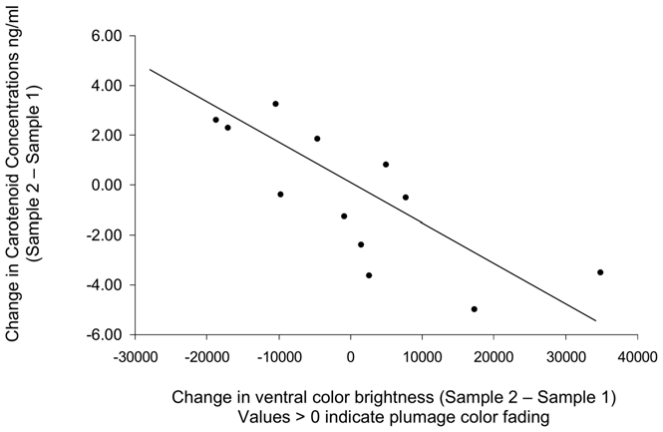
Seasonal changes in feather color are associated with changes in circulating carotenoid concentrations. Evidence of concomitant changes in carotenoids and ventral color suggesting a dynamic relationship between morphological and physiological traits: melanin-based color fading is strongly associated with a decline in carotenoid concentration in both males and females. N = 12 individuals for whom we had both two sets of carotenoid and feather samples.

Finally, we uncovered intriguing concomitant relationships in both males and females between changes in plasma carotenoids and changes in plumage brightness [Figure 2: changes in carotenoid balance as a function of changes in throat brightness: F_1, 6.29_ = 6.70, P<0.05, controlling for gender: (F_1,6.04_ = 1.50, P>0.26), days between feather and plasma samples because melanin-based color is known to change over time: (F_1, 6.09_ = 2.97, P>0.14), and *site* differences, adjusted R-squared = 0.92]. Although body mass is another variable that changes within individuals over time, we did not uncover a correlation between changes in body mass and changes in carotenoid concentrations over time (F_1,20.24_ = 0.11, P>0.74, controlling for gender: (F_1,19.85_ = 2.57, P>0.13), days between sampling events (F_1,20.31_ = 1.11, P>0.30), and *site* differences).

### Temporal Changes in Carotenoids, Not Data from Single Point Samples, Correlate with Reproductive Performance

**Figure 3:**
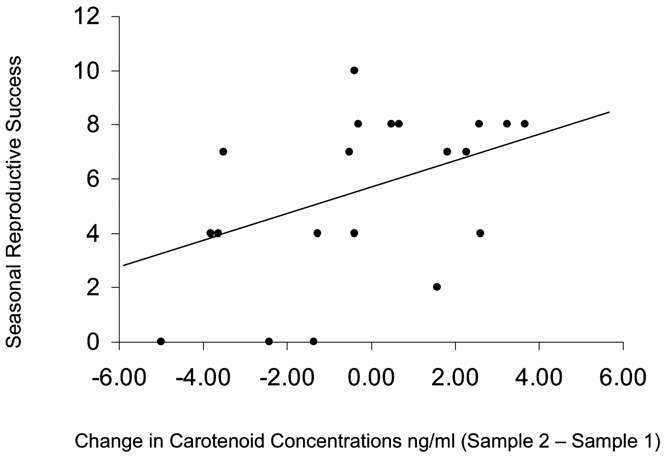
Seasonal balance in circulating concentrations predicts variation in annual reproductive performance. Controlling for variation in clutch initiation dates, individuals who maintain high levels of circulating carotenoids have greater reproductive success. N = 27 individuals.

Although two successive carotenoid measures were positively correlated within individuals [F_1, 15.96_ = 7.55, P<0.02, controlling for gender: (F _1,15.85_ = 1.93, P>0.18), mass: (F_1, 16.54_ = 3.85, P>0.06), throat brightness: (F_1, 16.16._ = 2.15, P>0.16) and *site* differences, adjusted R-squared  = 0.53], neither of these two temporally separate measures significantly predicted reproductive performance over the span of the four-month breeding season of this species (sample 1: F _1, 55.41_ = 0.01, P>0.94, controlling for gender (F _1, 53.46_ = 0.03, P>0.95) and *site* differences; sample 2: F _1, 19.93_ = 3.81, P>0.10, controlling for gender (F _1, 19.82_ = 0.05, P>0.82) and *site* differences). Instead, controlling for variation in clutch initiation date (and thus, breeding schedule), the change in carotenoid levels of individuals over our two sampling points was strongly and positively tied to seasonal reproductive success (total number of fledged young) even when accounting for variation in coloration (Table 3; Figure 3).

## Discussion

In our longitudinal field study of carotenoid circulation and breeding in barn swallows, we found that single-time-point estimates of carotenoid concentration were not significantly correlated with reproductive performance. Instead, we found that males and females who maintained high concentrations of these molecules over time were heavier in body mass and had greater seasonal reproductive success compared to individuals whose carotenoid concentrations declined during the breeding season. Moreover, we uncovered a strong relationship between changes in ventral coloration and carotenoid concentrations, suggesting that these variables are linked. These results are the first to elucidate patterns of temporal variation in carotenoid concentrations in a natural and functional reproductive context, but see [9] for an analysis of carotenoid change as a function of migration arrival date in barn swallows, and further suggest an important yet overlooked coupling between physiological and morphological variation [23].

Reproduction is thought to be one of the most somatically costly life-history traits and induces oxidative stress in many animals [24]–[26]. However, an experimental approach in captivity has been taken in nearly all of the work on this subject, and the role of individual variation is commonly overlooked [27]. Indeed, it is becoming increasingly clear that individuals strongly vary in their ability to respond to internal and external sources of stress and that this fascinating source of variation has enormous implications for the interplay between physiology, morphology and life history evolution [3].

In our correlational study, we obviously cannot ascribe causal effects of such circulating carotenoid changes on offspring output *per se*. However, longitudinal studies such as ours that clearly demonstrate the important dynamics of physiological underpinnings of reproductive performance are essential for motivating future experimental studies and addressing the limitations of current data sets. Of course, it is important to consider the potential sources of variation in carotenoid accumulation as they may pertain to reproductive performance. Prior studies of birds have shown that elevated carotenoid accumulation can improve maternal egg production [7] as well as egg-yolk conditions for embryonic development [28], but here we show a longer- and later-term association between body carotenoid supplies and reproduction. Previous analyses of barn swallows from two geographically separate populations in North America demonstrate that females paired to darker males (shown here to have greater circulating concentrations of carotenoids) fed shared offspring at a greater rate [29]. It is therefore plausible that relationships between ventral color and reproductive success [17] are to some extent mediated by parental carotenoid status. Interestingly, additional variables related to fecundity are not significantly correlated with carotenoid status, including clutch initiation date, clutch size, or the probability of multiple broods (RJS unpubl. data), suggesting that differential allocation of parental care may be important for explaining our results.

Our results are consistent with the hypothesis that physiological underpinnings of traits like ventral color, body mass, carotenoids, and reproduction are highly dynamic and that the positive management of physiological regulators (e.g. hormones, carotenoids) is adaptive [23], [30]. Moreover, dynamical relationships between morphological and physiological traits are critical to account for when calculating the costs of both developing and expressing morphological signals used in competitive and reproductive contexts. Although previous research has demonstrated seasonal changes in melanin-based plumage in barn swallows [31], the interesting linkage between seasonal changes in coloration and carotenoid concentrations suggest that some individuals are both better at maintaining feather coloration and acquiring carotenoids via diet–possibly due to abilities to dedicate time both for preening and efficient foraging. Whereas previous studies have relied on single samples from individuals, our results suggest that repeated sampling schemes are perhaps best for gaining more comprehensive information about individual-variation in carotenoids in an ecological and evolutionary context.

## References

[1] Hughes DA (2001). Dietary carotenoids and human immune function.. Nutrition.

[2] Krinsky NI (2001). Carotenoids as antioxidants.. Nutrition.

[3] Alonso-Alvarez C, Bertrand S, Devevey G, Gaillard M, Prost J (2004a). An experimental test of the dose-dependent effects of carotenoids and immune activation on sexual signals and antioxidant activity.. Am Nat.

[4] Hõrak P, Saks L, Zilmer Z, Karu U, Zilmer K (2007). Do dietary antioxidants alleviate the cost of immune activation? An experiment with greenfinches.. Am Nat.

[5] Costantini D, Møller AP (2008). Carotenoids as minor antioxidants for birds.. Funct Ecol.

[6] McGraw KJ, Ardia DR (2003). Carotenoids, immunocompetence, and the information content of sexual colors: an experimental test.. Am Nat.

[7] Blount JD, Metcalfe NB, Birkhead TR, Surai PF (2003). Carotenoid modulation of immune function and sexual attractiveness in zebra finches.. Science.

[8] Pike TW, Blount JD, Bjerkeng B, Lindstrom J, Metcalfe NB (2007). Carotenoids, oxidative stress and female mating preference for longer lived males.. Proc Roy Soc B.

[9] Ninni P, de Lope F, Saino N, Haussy C, Møller AP (2004). Antioxidants and condition-dependence of arrival in a migratory passerine.. Oikos.

[10] Catoni C, Peters A, Schaefer M (2008). Life history trade-offs are influenced by the diversity, availability, and interactions of dietary antioxidants.. Anim Behav.

[11] Costantini D, Verhulst S (2009). Does high antioxidant capacity indicate low oxidative stress?. Funct Ecol.

[12] Wiersma P, Selman C, Speakman JR, Verhulst S (2004). Birds sacrifice oxidative protection for reproduction.. Proc Roy Soc B.

[13] Bertrand S, Alonso-Alvarez C, Devevey G, Faivre B, Prost J (2006). Carotenoids modulate the trade-off between egg production and resistance to oxidative stress.. Oecologia.

[14] Monaghan P, Metcalfe N, Torres R (2009). Oxidative stress as a mediator of life history trade-offs: mechanisms, measurements, and interpretation.. Ecol Lett.

[15] Saino N, Stradi R, Ninni P, Møller AP (1999). Carotenoid plasma concentration, immune profile, and plumage ornamentation of male barn swallows (*Hirundo rustica*).. Am Nat.

[16] McGraw KJ, Safran RJ, Evans MR, Wakamatsu K (2004). European barn swallows use melanin pigments to color their feathers brown.. Behav Ecol.

[17] Safran RJ, McGraw KJ (2004). Plumage coloration, not length or symmetry of tail-streamers, is a sexually selected trait in North American barn swallows.. Behav Ecol.

[18] Safran RJ, Neuman CR, McGraw KJ, Lovette IJ (2005). Dynamic paternity allocated as a function of male plumage color in barn swallows.. Science.

[19] McGraw KJ, Tourville EA, Butler MW (2008). A quantitative comparison of the commonly used methods for extracting carotenoids from avian plasma.. Behav Ecol Sociobiol.

[20] Safran RJ, Pilz KM, McGraw KJ, Correa S, Schwabl H (2008). Maternal deposition of egg-yolk androgens and carotenoids in barn swallows: is allocation related to indicators of parental quality?. Behav Ecol Sociobiol.

[21] Safran RJ (2004). Adaptive site selection rules and variation in group size of barn swallows: individual decisions predict population patterns.. Am Nat.

[22] Safran RJ (2006). Nest-Site Selection Decisions in Barn Swallows: What Predicts Reproductive Performance?. Can J Zool.

[23] Safran RJ, Adelman J, McGraw KJ, Hau M (2008). Sexual signal exaggeration affects physiological state in a social vertebrate.. Curr Biol.

[24] Wang Y (2001). A cost of reproduction: oxidative stress susceptibility is associated with increased egg production in Drosophila melanogaster.. Exp Gerontol.

[25] Alonso-Alvarez C, Bertrand S, Devevey G, Prost J, Faivre B (2004b). Oxidative stress as a proximate cost of reproduction.. Ecol Lett.

[26] Dowling DK, Simmons LW (2009). Reactive oxygen species as universal constraints in life-history evolution.. Proc Roy Soc B.

[27] Ardia DR (2005). Individual quality mediates tradeoffs between reproductive effort and immune function in tree swallows.. J Anim Ecol.

[28] Saino N, Ferrari R, Romano M, Martinelli R, Møller AP (2003). Experimental manipulation of egg carotenoids affects immunity of barn swallow nestlings.. Proc R Soc Lond B.

[29] Maguire SE, Safran RJ (2010). Morphological and genetic predictors of parental care in the North American barn swallow.. J Avian Biol.

[30] Rubenstein DR, Hauber ME (2008). Dynamic feedback between phenotype and physiology in sexually selected traits.. Trends in Ecol and Evol.

[31] Hasegawa M, Arai E, Watanabe M, Nakamura M (2008). Methods for correcting plumage color fading in the Barn Swallow.. Ornithol Sci.

